# Access to PrEP and other sexual health services for cisgender women in the United States: a review of state policy and Medicaid expansion

**DOI:** 10.3389/fpubh.2024.1360349

**Published:** 2024-06-25

**Authors:** Ashley Chory, Keosha Bond

**Affiliations:** ^1^Arnhold Institute for Global Health, Icahn School of Medicine at Mount Sinai, New York, NY, United States; ^2^New York Medical College, Valhalla, NY, United States; ^3^City University of New York School of Medicine, New York, NY, United States; ^4^Yale University School of Public Health, New Haven, CT, United States

**Keywords:** PrEP, women, sexual and reproductive health, Medicaid, policy, United States

## Abstract

Pre-exposure prophylaxis (PrEP) has the potential to prevent new HIV infections, but it is unclear how state policies governing sexual and reproductive health services (SRH) impact access for cisgender women. The objective of this review is to identify barriers to PrEP access for cisgender women in the United States. Using the CDC Atlas Program, 20 states with the highest HIV incidence among cisgender women were included in this analysis. Through a search conducted in May–July 2022 of CDC, PrEPWatch.org, and other State Department and Insurance websites, Medicaid expansion status, pharmacist PrEP prescribing laws, financial support programs, and Traditional Medicaid coverage of PrEP, HIV testing, and emergency contraception were reviewed. Of the included states, nearly half did not expand Medicaid at the state level. Emergency contraception and HIV testing was covered under Traditional Medicaid for almost all included states, but insurance stipulations and eligibility requirements remain. Although PrEP is covered under all Traditional Medicaid plans, six states require pre-authorization. Three states have HIV testing mandates, four allow pharmacists to prescribe PrEP and six have financial support programs to cover the cost of PrEP. Medicaid expansion, pre-authorization requirements for PrEP prescriptions and emergency contraception, and limitations on pharmacist prescribing abilities were identified as barriers to SRH access for cisgender women. Medicaid expansion should be prioritized as an approach to expanding access to HIV prevention services at the state level.

## Introduction

Legislation and other government policies impact the way in which individuals interact with the health care system, in many cases reducing access to critically needed services (1, 2). This is especially the case in sexual and reproductive health and HIV care, where women and other birthing persons face restrictions on sexual education and reproductive rights due to limitations on insurance expansion; these limitations perpetuate cycles of poor access to and retention in care, and high rates of unintended pregnancy and HIV transmission. These restrictive laws are informed by, and perpetuate mass incarceration, poverty, racism, homophobia, inequitable gender norms and other inequities that facilitate the risk-taking behaviors that contribute to increased HIV incidences among vulnerable populations (3). These factors increase the susceptibility of cisgender women to HIV infection and worsen their long-term health outcomes (2).

In 2019, there were 36,801 new HIV diagnosis in the United States, of which 19% were among cisgender women for whom heterosexual contact is the dominant mode of acquisition (4). Racial disparities in HIV among US women are stark; 93% of new HIV infections among Black women would not have occurred if incidence were the same for Black as for White women (5). The HIV mortality rate is nearly six per 100,000 among Black women, compared with 0.3 among White women (6). There are several challenges that contribute to cisgender women’s vulnerability to HIV infection; factors like racism, discrimination, and HIV-stigma influence access to and the quality of care received by women, particularly women of color; higher risks of exposure due to engagement in receptive sex; and inequitable gender norms that contribute to intimate partner violence and imbalances in health decision making power (7). Additionally, many women may not perceive their risk to be high due to their relationship status with male partners that are expected to be monogamous. HIV infection can be prevented by HIV pre-exposure prophylaxis (PrEP), a medication that is ~62–84% (8–11) efficacious among women when taken consistently. Yet, in the US, PrEP is prescribed to just 2% of the ~468,000 women whose main mode of acquisition is heterosexual sex (12). While much of the marketing for PrEP has targeted men who have sex with men (MSM) and transgender individuals, cisgender women may also benefit from its’ use (10, 11). To achieve the goal of ending the HIV epidemic, it is critical that we increased use and acceptability of PrEP among cisgender women.

Despite data on PrEP efficacy, in 2020, only 10% of cisgender women who could benefit from PrEP were prescribed it in the United States (4). When these data are evaluated by racial group, the differences are even more concerning; Black and Hispanic/Latino individuals represent the group for whom PrEP is recommended but have the lowest rates of use among all racial/ethnic groups; preliminary CDC data show only 9% (42,372) of the nearly 469,000 Black people and only 16% (48,838) of the nearly 313,000 Hispanic/Latino people who could benefit from PrEP received a prescription in 2020 (13). These data highlight the racial and gender inequities that drive the HIV epidemic; there is a critical need to address the root causes, particularly the laws and policies that facilitate these disparities, including poverty, housing instability, unequal access to health care, lack of education, stigma, and systemic racism. The consideration of these intersections are timely, as the UNAIDS Global AIDS Strategy for 2021–2026 is particularly focused on addressing the inequities that drive the AIDS epidemic and is situated within the UN Sustainable Development Goals that guide policy decision making (14).

There have been several policy changes under the Affordable Care Act to support usage of PrEP. In June 2019, a national panel of health experts concluded that PrEP was a crucial tool in fighting the AIDS epidemic. The decision to classify PrEP as an effective preventative service prompted rules requiring health insurance to cover the expenses under the terms of the Affordable Care Act; insurance companies were required to comply with this order by January of 2021. The Department of Labor amended these guidelines in July 2021 after facing opposition from the insurance industry, which stated that patients should not be charged for medical services related to a PrEP prescription, including doctor visits and laboratory tests. The states that extended their Medicaid programs under the Affordable Care Act and those that provided programs to defray the costs of PrEP benefited from greater usage of the preventative modality (15). In other words, if PrEP is available for free or at a reduced cost, more people utilize it. Uptake of PrEP for HIV prevention has significant cost savings implications for both insurance companies and the health system overall, as an evaluations in the United States indicate a lifetime savings of over $200,000 USD for each HIV infected averted by PrEP use (16).

Many uninsured women are eligible for insurance coverage but are not enrolled. In 2020, one million women were in the “Medicaid coverage gap,” which affected one in every five (2.1 million) uninsured women who qualified for Medicaid but were not enrolled (17). These women remain ineligible for Medicaid because they live in a state that has not extended its Medicaid program, but are eligible for Health Insurance Marketplace subsidies which helps to lower or eliminate the out-of-pocket cost of monthly premiums for health coverage (18) because their income is less than the lower income limit (100% FPL) (17). Medicaid expansion is linked to an array of health benefits, including more equitable access to PrEP and drug assistance programs to help fill gaps and cover costs (17). In 2018, 20% of people living with HIV (PLWH) lacked health insurance in non-expansion states, compared to 6% in expansion states; Medicaid coverage was more prevalent in expansion states (46% vs. 30%) among the states studied (17). This review explores attributes of state-level laws and programs that may impact access to PrEP for cisgender women in a sample of U.S. states with higher HIV incidence. Attributes of interest include Medicaid coverage of key sexual health services, PrEP prescription requirements, and financial support programs related to service acquisition.

## Methods

This study included a review of state-level laws and programs that govern and impact the accessibility of PrEP and other SRH services for cisgender (women assigned female at birth and currently identify as female) women in the United States.

### Inclusion criteria

Using the CDC Atlas Plus program, data on new HIV diagnoses among cisgender women by state were obtained on May 4, 2022. We used the 2019 CDC dataset as it was the latest, most comprehensive dataset available. The 20 U.S. States and Territories with the highest rates of new HIV infections among cisgender women in 2019 were included in this analysis.

### Search strategy

We conducted an internet search for state policies related to sexual and reproductive health access for cisgender women using the following key words: [“Pre-exposure prophylaxis OR PrEP”] AND [“Policy” OR “Strategies” OR “Guidelines”] AND [“Women” OR “Girls”]. We specifically targeted reports by the CDC and PrEPwatch.org, a website that tracks the global availability of PrEP and ongoing medication trials. We searched the official websites of State Departments of Health and of state governments responsible for regulating access to medical interventions (including prevention, testing and/or screening, and treatment) to identify policies related to PrEP and women’s rights to sexual and reproductive health services. Our policy search was conducted from inception until 21 July 2022.

### Data extraction

The following variables were extracted and compiled for the included states: Medicaid expansion status, PrEP pre-authorization requirements, pharmacist PrEP prescribing laws, PrEP financial support programs, and Traditional Medicaid coverage of the following services: PrEP, HIV testing, emergency contraception and coverage status without a prescription. These data were documented in tabular form in Microsoft Excel for analyses. The research team met to discuss findings and exchange information, and adjusted search strategies as necessary. We defined Medicaid expansion status as implementing Medicaid expansion before June 1, 2022 (18).

## Results

The following U.S. states represent the states and territories with the highest HIV incidence among cisgender women in 2019, in order from highest to lowest: District of Columbia, Georgia, Maryland, Florida, Louisiana, Mississippi, Texas, Alabama, West Virginia, New Jersey, Delaware, South Carolina, Nevada, New York, North Carolina, Arkansas, Puerto Rico, Tennessee, Massachusetts, Illinois. Table 1 shows the included states and key domains of inquiry.

**Table 1:** U.S. state policies related to sexual and reproductive health coverage.

		Coverage under traditional Medicaid							
State (by rate per 100,000, high to low)	Medicaid expansion state	PrEP	Requires pre-authorization	HIV test	Emergency contraception	Prescription needed	HIV testing mandate	Pharmacists prescribe PrEP	State financial support programs
District of Columbia	Yes	Yes	Yes	Yes	Yes	Yes	No, all insurance covers testing in the ED	No	Yes
Georgia	No	Yes	No	Yes	No	N/A	No	No	No
Maryland	Yes	Yes	Yes	Yes	Yes	Yes	No	Bill pending	No
Florida	No	Yes	No	At-risk only	Yes	No, minimum age 12	No	Yes	No
Louisiana	Yes	Yes	No	Yes	Yes	No	No	No	No
Mississippi	No	Yes	Yes	Yes	No	N/A	No	No	No
Texas	No	Yes	No	Yes	Yes	No	No	No	No
Alabama	No	Yes	No	Yes	Yes	No, req. prior authorization	No	No	No
West Virginia	Yes	Yes	No	Yes	Yes	No	No	No	No
New Jersey	Yes	Yes	Yes	Yes	Yes	Yes	No	Bill pending	No
Delaware	Yes	Yes	Yes	Yes	Yes	No	Routine opt-out testing	No	No
South Carolina	No	Yes	Yes	Yes	Yes	No	No	No	No
Nevada	Yes	Yes	No	Yes	Yes	No	Yes	Yes	No
New York	Yes	Yes	Yes	Yes	Yes	Yes	Yes	Bill pending	Yes
North Carolina*	No	Yes	No	Yes	Yes	No, prescription pharmacy benefit only	No	Yes	Yes
Arkansas	Yes	Yes	No	Yes	Unclear	Unclear	No	No	Yes
Puerto Rico	Yes	Yes	No	Yes	Unclear	Unclear	No	No	No
Tennessee	No	Yes	No	Yes	Yes	No	No	Yes	No
Massachusetts	Yes	Yes	No	Yes	Yes	No	No	Bill pending	Yes
Illinois	Yes	Yes	No	Yes	Yes	Yes	Yes	Bill pending	Yes

* Medicaid coverage under expansion in North Carolina began on December 1, 2023 after this analysis was completed.

### Medicaid expansion and coverage

Table 1 shows the state Medicaid expansion status and care coverage under Traditional Medicaid. Of the 20 states with the highest HIV incidence among cisgender women, almost half did not expand Medicaid at the state level. Although PrEP is covered under all Traditional Medicaid plans, six of the included states require pre-authorization, representing a barrier to care initiation. HIV testing for all populations was covered by Traditional Medicaid in all states, except for Florida, which dictates that an individual must be considered ‘high risk’ per CDC guidelines to receive HIV testing. CDC guidelines list the following HIV risk behaviors for cisgender women: in the past 12 months, had sex without using any HIV prevention strategy (had sex with a partner whose status was unknown, or was HIV positive and not virally suppressed; had sex without using a condom; had sex with someone who was not taking PrEP) and/or used a syringe or any other injection equipment after someone else used it.

Emergency contraception was covered under Traditional Medicaid for almost all states included in this analysis; Georgia and Mississippi do not cover these services, and data on Arkansas and Puerto Rico remain unclear. Of the 16 states that covered emergency contraception, DC, Maryland, New Jersey, New York, and Illinois require a prescription, Alabama requires prior authorization and North Carolina requires beneficiaries to be enrolled in the prescription benefit plan for insurance coverage to be used. While a prescription is not needed in Florida, emergency contraception benefits are only accessible for individuals 12 years and older. In summary, only 8 of the 16 states that cover emergency contraception have no insurance related barriers to receipt.

### Other policies influencing PrEP accessibility

State-level HIV testing mandates and pharmacist PrEP prescribing capabilities were also reviewed. Only three of the included states, New York, Nevada, and Illinois had HIV testing mandates that require primary care and emergency providers to offer HIV testing to all patients. A fourth state, Delaware, has implemented an opt-out program that integrates HIV testing into routine laboratory testing, and requires patients to intentionally opt-out of that lab test. These approaches aim to expand accessibility and normalize and reduce the stigma around HIV testing. While the District of Columbia does not have a HIV testing mandate, they have required all insurers to cover the cost of HIV testing in the Emergency Department, a step in expanding access to screening. Florida, Nevada, North Carolina, and Tennessee have passed legislation to allow pharmacists to prescribe PrEP; prescriber training requirements are included in Table 2. Five additional states, Maryland, New Jersey, New York, Massachusetts, and Illinois have bills pending that would expand PrEP prescribing access to Pharmacists.

**Table 2:** Pharmacist PrEP prescriber requirements.

State	Legislation	Education requirements	Prescribing criteria
Florida	House Bill 389 Expands pharmacy practice to include certain drug therapy services, including PrEP Under Collaborative Pharmacy Practice Agreement (CPPA), testing and treatment under a written protocol with a supervising physician	Doctor of Pharmacy Degree or 5 years of experience as a licensed pharmacist License renewal every 2 years with an 8-h continuing education course 20-h course: performance of patient assessment; ordering, performing, and interpreting clinical and laboratory tests	Professional liability insurance coverage Patient records medical system for at least 5 years
North Carolina	House Bill 96 Under CPPA and standing order from collaborating physician, may dispense without a prescription Pharmacists must counsel patients and notify primary care provider of PrEP usage	Doctor of Pharmacy degree Bachelor of Science in Pharmacy Completed two NCCPC or ACPE approved certificate programs Completion of an ASHP accredited residency program	CPPA with a physician licensed in NC who has prescribing authority, including of controlled substances, approved by the North Carolina Board of Pharmacy 5 years of clinical experience
Tennessee	Tennessee Board of Pharmacy Rule 1140–03-0.17 (5)(b) Under CPPA and standing order from collaborating physician, may dispense PrEP without a prescription Pharmacists must counsel patients and notify their primary care provider of PrEP usage	Doctor of Pharmacy degree OR Bachelor of Science degree in pharmacy and in active practice Pass the NAPLEX and MPJE Licensed by Tennessee Board of Pharmacy	CPPA with a physician, that includes guidelines for treatment, screening, and preventative care CPPA is renewed and updated every 2 years
Nevada	Nevada Senate Bill 325 Pharmacists may prescribe, dispense, and administer PrEP Requires all state regulated health plans, including Medicaid and state employee plans, to provide coverage and reimbursement for medications and related pharmacist clinical services at a rate equal to other practitioners	Doctor of Pharmacy degree Pass the NAPLEX and the MPJE Licensed by Nevada Board of Pharmacy	Complete a two-hour education course approved by ACPE Liability insurance coverage of 1 million dollars Pharmacist must complete patient HIV screening assessment and counseling on proper use of medication

### PrEP financial support programs

National and State-level PrEP financial aid programs, eligibility and benefits were reviewed. Seven national financial support programs were identified; three programs were established by pharmaceutical companies, Gilead Sciences, a Biotechnology company that researchers and develops antiviral drugs, and is creator of Truvada for PrEP, and ViiV Healthcare Limited, a joint venture by Pfizer and GlaxoSmithKline. The remaining programs were established by Foundations providing financial assistance for medical expenses. In addition to the national level programs, six states have established financial support programs to cover the cost of PrEP. Lastly, two telemedicine programs offering comprehensive PrEP care, including laboratory screenings were identified. Interestingly, one company offers two different programs, one specifically targeting cisgender women. Program details, patient eligibility and benefits are described in detail in Table 3.

**Table 3:** PrEP financial support programs.

Location	Program name	Application criteria (location, income, insurance)	Benefits
National	Gilead Sciences Advancing Access Patient Assistance Program	Income at or below 500% FPL Uninsured or underinsured	Co-payment assistance, reimbursement support, and patient assistance programs
National	Gilead Advancing Access Cost Sharing Assistance Program	Income at or below 500% FPL Uninsured or underinsured	Covers prescription costs for Truvada and Descovy
National	The Patient Advocate Foundation	Reside and receive treatment in the U.S. Income at or below 300% FPL Accepts all insurance, must cover pharmaceutical products	Maximum annual assistance: $7,500 to cover the costs of clinical visits, co-insurance, and deductibles.
National	Ready, Set, PrEP	Reside in the U.S., including tribal lands/territories No income eligibility requirement, For individuals who lack prescription drug coverage	Provides free, oral PrEP medication
National	Patient Access Network Foundation	Reside in the U.S., including territories, Income at or below 500% FPL Medicare insurance with prescription benefit	Maximum annual assistance: $3,600 to cover the costs of out-of-pocket medication costs, co-pays, and health insurance premiums
National	My Good Days	U.S. Social Security number required, receive treatment in the U.S. Income at or below 500% FPL Medicare, or Military Insurance	Maximum annual assistance: $7,500 to cover co-pays
National	ViiV Connect	U.S., DC, and Puerto Rico Income less than 500% FPL Not eligible for Medicaid/Mi Salud; no prescription drug coverage. Have Medicare Part B, D, or Advantage Plan, and spent $600+ on out-of-pocket prescription expenses that year	Provides free, long-acting injectable PrEP
Telemedicine	Mistr	Uninsured or underinsured men who have sex with men	Free provider consultation, laboratory tests, PrEP prescription
Telemedicine	Sistr	U.S., DC., and Puerto Rico. Uninsured or underinsured women	Free provider consultation, laboratory tests, PrEP prescription
District of Columbia	DC Health Drug Assistance Program	DC Metropolitan Area Resident Income at or below 500% FPL Have insurance Provider declaration of high risk for HIV infection	Provides PrEP medication
New York	PrEP Assistance Program	New York Resident Income at or below 500% of FPL Uninsured	Covers costs of clinical visits and lab testing; does not cover cost of PrEP medication
North Carolina	Western North Carolina AIDS Project	Uninsured or underinsured	Provides copay assistance and PrEP medication at a discount, or for free depending on eligibility
Arkansas	AR Care	Uninsured	Maximum annual assistance: $2,5000 to cover the costs of clinical visits, and prescriptions
Massachusetts	PrEP Drug Assistance Program	Live in Massachusetts Income at or below 500% FPL	Covers out of pocket costs for those with health insurance. Covers the full cost of PrEP for uninsured.
Illinois	Illinois PrEP Assistance Program	High risk for HIV infection per CDC guidelines	Client navigation for PrEP services: Education, Medication Access, provider referrals, enrollment into payment assistance programs
Tennessee	AIDs Drug Assistance Program	Tennessee State Residency Income at or below 400% FPL	PrEP medication and insurance financial assistance

## Discussion

This analysis of 20 U.S. states and territories with high HIV incidence among cisgender women reviewed sexual and reproductive health policies that may represent barriers to receipt of care. While PrEP is covered under Traditional Medicaid nationwide, barriers related to pre-authorization requirements, cost, and provider accessibility remain; only four of the included states have passed legislation to allow pharmacists to prescribe the HIV prevention medication and only six states have financial support programs available. Similar barriers were identified for emergency contraception and HIV testing, which were not covered by Traditional Medicaid in all included states.

### Medicaid expansion and service utilization

In 2022, states with expanded Medicaid programs had 1.4 times higher PrEP use rates compared to those without expansion (19) (AIDvu). State-led Medicaid expansion under the Affordable Care Act expanded insurance coverage to nearly all adults with incomes up to 138% of the Federal Poverty Level, expanded parent coverage of dependents until the age of 26, and provided states with an enhanced federal matching rate for their expansion populations (18). These changes were critical in providing health insurance, and for increasing accessibility of resources across the health system.

Several studies have investigated the relationship between Medicaid expansion and PrEP use. Previous work to compare care coverage and utilization among MSM found that MSM in states that did not expand Medicaid were less likely to have insurance, utilize health care or access PrEP; MSM in expansion states were more likely to use PrEP (20). Additionally, they found that 20% of HIV positive and 30% of HIV negative MSM in non-expansion states were uninsured (20). In an additional analysis exploring PrEP uptake by MSM and transgender individuals, Carneiro and colleagues found that individuals in states without Medicaid expansion had 31% lower odds of being current PrEP users (aOR = 0.69, 95% CI 0.54–0.88), than individuals living in states with full expansion (aOR = 0.73, 95% CI 0.56–0.95) (21). This data is further complicated by gender identity; those who identified as female or as a transgender person had 66 and 29% significantly lower odds of being current PrEP users than those identifying as male (21). These findings are consistent with uptake of HIV testing, as Medicaid expansion has been shown to be associated with significant increases in testing (22–24).

These data highlight the positive relationship between access to health insurance and care uptake, yet there are still challenges for women when it comes to persistent PrEP use (the length of time with consistent refills) and the uptake of critical sexual health services (25). While it has been noted that commercially insured persons have a longer period of PrEP persistence than Medicaid insured persons, there are also some differences in sex (26). One year after starting PrEP, 21% of women with Medicaid insurance continued taking it, compared to 32% of men; PrEP persistence for women was 5.8 months compared to 7.1 months for men (26). While there is little information on the causes of these disparities in persistence, several explanations have been put forth, including varying degrees of HIV and PrEP -related stigma, limited access to healthcare, financial limitations, or less encouragement to continue using PrEP by their community or healthcare provider. Under the Affordable Care Act, preventive services, like HIV testing, remain covered at no out-of-pocket cost, which helps remove financial barriers to screening and facilitates increased engagement in the health system (23). Following Medicaid expansion in New York, PrEP prescriptions among Medicaid receipts increased (27); this suggests that for many key populations, insurance coverage remains a barrier to uptake of PrEP services. In this analysis we found that eight of the top 20 states for new HIV infections chose not to expand Medicaid. The South accounts for most new HIV diagnoses among cisgender women (50%), yet seven out of 14 states continue to opt of adoption of Medicaid expansion (Medicaid coverage under expansion in North Carolina began on December 1, 2023) (18). In 2020, the PrEP-to-demand ratio for cisgender women in the South was twice as low as in other regions, despite having the highest number of cisgender women PrEP users. This suggests a significant unmet demand for PrEP among cisgender women in the South (19, 28).

This is not surprising, as individuals at high risk for HIV may not have insurance coverage and therefore not be able to access prevention services; this is further complicated by barriers related to cost and access to a prescribing provider.

### PrEP cost and prescriber requirements

PrEP cost and accessibility remain key barriers to acquisition and retention in care for individuals at high risk for HIV infection. Under the Affordable Care Act, PrEP medication, clinic visits and associated laboratory tests are free under most insurance plans; without insurance, however, the totality of cost is in the tens of thousands of dollars per year. We summarized the national level programs available to cover the costs for uninsured individuals, as well as those insured with other gaps in coverage. Only six of the included states in this analysis have established additional financial support programs to cover the costs of PrEP and associated clinic and laboratory costs; all but one of those states is a Medicaid expansion state, further highlighting the gap in financial support for individuals in non-expansion states.

Currently any licensed prescriber can prescribe FDA approved formulations of PrEP, regardless of specialization status in infectious disease or HIV medicine. Despite this, studies report limited knowledge, prescribing and insurance coverage concerns, and discomfort among physicians as barriers to providing PrEP to their patients (29–31). Primary care facilities may be the most appropriate entry point for otherwise healthy individuals at high risk for HIV, and yet there remain barriers to access to providers willing and comfortable prescribing the medication.

Provision of HIV prevention services at the pharmacy represents one approach to bridging the gap to PrEP initiation, as pharmacies remain much more accessible to the general population than other health care touch points; it also eliminates an additional stop in the care continuum, as patients can complete PrEP screening and pick up their medications in the same location. Under new PrEP expansion programs, pharmacists can order an HIV and other baseline testing requirements for PrEP initiation, and then schedule a consultation for counseling and initiation of PrEP upon receipt of the results. Pharmacist-prescribed PrEP is often limited to a 2–3-month supply, after which a patient would require a prescription from a non-pharmacist, license provider; this process may facilitate a more accessible and rapid initiation process until a relationship with a longer-term provider can be established.

A recent scoping review (32) was conducted discussing pharmacy-based interventions to increase PrEP use in the United States; the authors report strong support among patients for pharmacist prescribed PrEP, provided a recommendation for greater collaboration between pharmacists and providers in HIV prevention, and evaluation models using collaborative practice agreements that show promise for PrEP initiation in pharmacies (32). Randomized control trials and comparisons of PrEP initiation between states with and without pharmacist prescribing authority need to be conducted to evaluate the impact of these policies. This data demonstrates potential for pharmacy-initiated PrEP to bridge an accessibility gap for people at high risk of HIV.

### Limitations

This review has limitations. All policies reviewed took place in the United States and its’ territories. While the dynamics of sexual and reproductive health policy that apply in the United States are particular, the effects of policy and insurance-based barriers to PrEP, emergency contraception and HIV testing are not limited to this setting. Additionally, only a subset of U.S. states were analyzed; while included states represent those with the highest burden of HIV infection among cisgender women, a broader set of state policies may improve generalizability.

## Conclusion

Cisgender women and birthing individuals remain a key population for HIV prevention and other SRH programming, especially following the U.S. Supreme Court’s decision to overturn Roe v. Wade and the subsequent implementation and enforcement of abortion laws starting in 2022.

Limited Medicaid coverage presents a substantial barrier to the extensive implementation of PrEP, as potential users frequently cite the financial burdens related to medication costs and healthcare visits as justifications for abstaining from adoption or maintenance of the PrEP regimen. The lack of health insurance continues to impede service utilization among individuals of reproductive age; therefore, further alternative initiatives such as pharmacy access and telemedicine are required to address the financial and accessibility disparities that continue to exist for this population segment. HIV testing mandates in emergency settings may additionally improve accessibility for screening and reduce associated stigma. Finally, revising prescribing requirements for key clinical areas may facilitate an important workforce expansion that will also support accessibility.

## Data availability statement

Publicly available datasets were analyzed in this study. This data can be found here: not applicable – CDC Atlas Data and Public Policy were used for this study.

## Author contributions

AC: Conceptualization, Formal analysis, Methodology, Project administration, Writing – original draft, Writing – review & editing. KB: Conceptualization, Supervision, Writing – review & editing.
